# Effects of Elevated Carbon Dioxide on Photosynthesis and Carbon Partitioning: A Perspective on Root Sugar Sensing and Hormonal Crosstalk

**DOI:** 10.3389/fphys.2017.00578

**Published:** 2017-08-08

**Authors:** Michael Thompson, Dananjali Gamage, Naoki Hirotsu, Anke Martin, Saman Seneweera

**Affiliations:** ^1^Faculty of Health, Engineering and Sciences, Centre for Crop Health, University of Southern Queensland Toowoomba, QLD, Australia; ^2^Faculty of Life Sciences, Toyo University Itakura-machi, Japan

**Keywords:** elevated carbon dioxide concentration (e[CO_2_]), sugar sensing and signaling, photosynthesis, hormone crosstalk, photosynthetic acclimation, carbon partitioning, hexokinase

## Abstract

Plant responses to atmospheric carbon dioxide will be of great concern in the future, as carbon dioxide concentrations ([CO_2_]) are predicted to continue to rise. Elevated [CO_2_] causes increased photosynthesis in plants, which leads to greater production of carbohydrates and biomass. Which organ the extra carbohydrates are allocated to varies between species, but also within species. These carbohydrates are a major energy source for plant growth, but they also act as signaling molecules and have a range of uses beyond being a source of carbon and energy. Currently, there is a lack of information on how the sugar sensing and signaling pathways of plants are affected by the higher content of carbohydrates produced under elevated [CO_2_]. Particularly, the sugar signaling pathways of roots are not well understood, along with how they are affected by elevated [CO_2_]. At elevated [CO_2_], some plants allocate greater amounts of sugars to roots where they are likely to act on gene regulation and therefore modify nutrient uptake and transport. Glucose and sucrose also promote root growth, an effect similar to what occurs under elevated [CO_2_]. Sugars also crosstalk with hormones to regulate root growth, but also affect hormone biosynthesis. This review provides an update on the role of sugars as signaling molecules in plant roots and thus explores the currently known functions that may be affected by elevated [CO_2_].

## Introduction

Since the industrial revolution, global atmospheric CO_2_ concentrations have rapidly increased, rising from 280 ppm to currently exceed 400 ppm (Canadell et al., 2007; Tans and Keeling, 2016). Predictions warn that the global CO_2_ concentration will continue to rise due in part to humanity's continued carbon emissions (Meehl et al., 2007). The resulting increase in CO_2_ will lead to a variety of both positive and negative effects on major agricultural crops used to feed the global population, many of which may yet be unknown. Elevated CO_2_ concentrations, written henceforth as e[CO_2_], cause increased photosynthesis in plants, which subsequently lead to positive effects such as greater growth, above-ground biomass, and yield (Ainsworth and Long, 2005; van der Kooi et al., 2016). However, e[CO_2_] also causes negative effects which could have serious consequences for the quality of the crop species, such as, declines in a variety of nutrients including protein concentrations of food crops (Fernando et al., 2015; Broberg et al., 2017), vitamins and some macro- and micro-elements (Högy and Fangmeier, 2008; Myers et al., 2014). Due to these negative effects, understanding plant responses to e[CO_2_] will become increasingly important as CO_2_ levels rise.

The increase in photosynthesis caused by e[CO_2_] results in an increase in carbohydrate production, which alters the plant's carbon and nitrogen metabolism. Apart from this direct effect on photosynthesis, many physiological processes are regulated indirectly, particularly via sugar sensing and signaling pathways. Sugar sensing and signaling plays an important role in the plant response to e[CO_2_], however, this is not well understood in relation to plant nutritional quality. Sugars are well known for their use as a source of energy and organic building blocks, and in plants they also play a role in regulating gene expression (Price et al., 2004), germination (Dekkers et al., 2004), and hormonal crosstalk (Mishra et al., 2009) among other functions.

Plant growth and development requires the uptake of soil nutrients by the roots, however, the concentration of nutrients in soil can vary and plants must adapt to the environment in order to fulfill their nutrient requirements. Sugars produced from photosynthesis are transported into roots where they can assist in regulating nutrient uptake via sugar sensing (Camañes et al., 2007; Lejay et al., 2008), though little research has been done in this area. How e[CO_2_] affects root function is not entirely understood, but we do know that it can affect the acquisition of soil nutrients (Taub and Wang, 2008; Pandey et al., 2015; Jayawardena et al., 2017). To what extent sugars may play a role in this is not currently known. This review aims to provide the current knowledge and understanding of sugar sensing in roots as well as the limited information available on how this is affected by e[CO_2_] in order to facilitate research into this area and safeguard crops from potential negative effects of future [CO_2_].

In order to study the effects of e[CO_2_] in the field, free-air CO_2_ enrichment (FACE) facilities have been established which allow plants to be grown in large scale open air environments. Utilizing either FACE or chamber experiments can affect the outcome of the experiment. For example, in comparison to FACE experiments, chamber studies using e[CO_2_] have been shown to further increase the yield of globally important food crops (Ainsworth et al., 2008). Plant growth differences between FACE and chamber experiments are likely influenced by the root growth, as restricting the available area for root growth reduces plant biomass (Poorter et al., 2012). Most of the studies discussed in this review were conducted with chamber experiments and to our knowledge no experiments have currently been done in FACE facilities for sugar sensing studies. As such, it is uncertain how the results of many of these sugar sensing studies will potentially change in plants grown in field conditions.

Many reviews have focused on various aspects of sugar sensing, however, this review discusses the limited amount of literature published on sugar signaling and sensing as it relates to plant root function, nutrient acquisition, and hormone crosstalk. As such, we have chosen roots as the focus of our review due to the current absence of reviews in this area, but more importantly due to their importance in determining the nutrient profile of plants. This review also discusses the effect of e[CO_2_] on the content of sugars in plants, including how photosynthesis and carbohydrate partitioning is affected, and how e[CO_2_] may affect sugar sensing in roots. The aim of this review is to provide the information necessary for scientists developing research projects involving sugar sensing in roots or the effect of e[CO_2_] on roots and sugar sensing.

## Elevated [CO_2_] and photosynthesis

**Figure 1:**
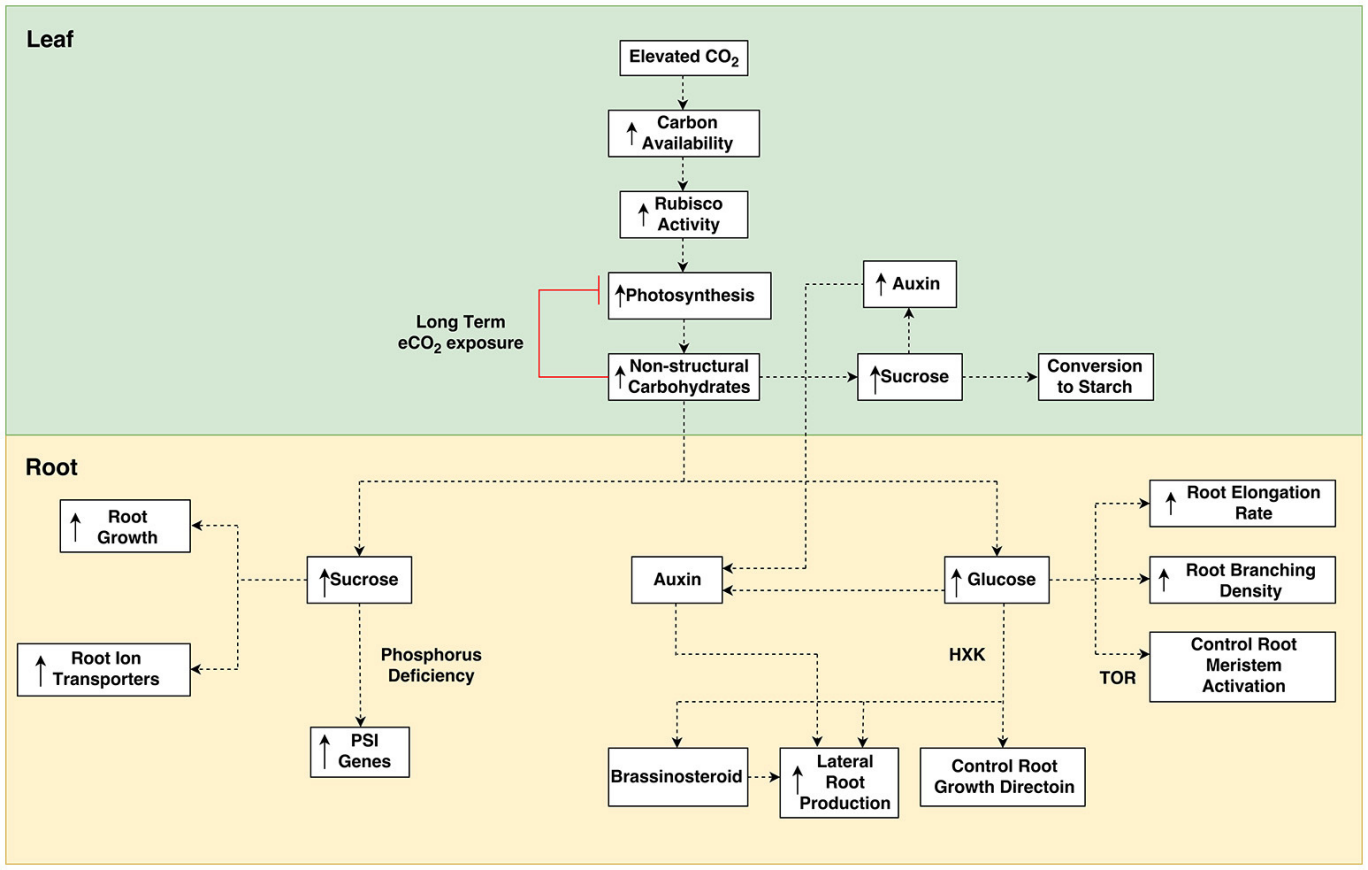
Overview of potential pathways for elevated [CO_2_] mediated sugar sensing responses. Elevated [CO_2_] increases the availability of carbon in leaves causing greater Rubisco activity and higher rates of photosynthesis. Greater photosynthesis increases the content of non-structural carbohydrates in leaves which can lead to greater starch reserves and increased auxin biosynthesis. Over long term e[CO_2_] exposure, photosynthesis is downregulated by increased carbohydrates. Carbohydrates are transported to roots, where they lead to greater root growth and stimulation of gene transcription. Root growth is also altered from the crosstalk of carbohydrates with hormones. HXK, Hexokinase; PSI, Phosphate Starvation Induced; TOR, Target-of-rapamycin.

Photosynthesis is a crucial process for controlling variables of crop growth and exposing C3 plants to e[CO_2_] generally increases photosynthesis (Drake et al., 1997; Ainsworth and Long, 2005; Wang et al., 2012; Figure 1). Increased photosynthesis under e[CO_2_] mainly occurs due to an increase in ribulose-1,5-bisphosphate (RuBP) carboxylase/oxygenase (Rubisco) activity. Rubisco catalyzes the carboxylation of RuBP, which is required for CO_2_ fixation, but also uses O_2_ as a substrate to oxygenate RuBP in a process called photorespiration (Makino and Mae, 1999). The carboxylation reaction of RuBP is not saturated at the current atmospheric [CO_2_], therefore, as the availability of CO_2_ increases under e[CO_2_] conditions so too will the rate of carboxylation (Drake et al., 1997). The other process, photorespiration, is wasteful in terms of energy, as it costs the plant more energy and does not lead to any gains in energy or carbon (Peterhansel et al., 2010). However, increasing the atmospheric CO_2_ levels increases the [CO_2_] surrounding Rubisco, shifting the ratio of CO_2_:O_2_ and thereby increasing the rate of carboxylation while decreasing the rate of oxygenation (Makino and Mae, 1999).

Despite the initial stimulation of photosynthesis seen at e[CO_2_], under long-term exposure to e[CO_2_] the plant incurs a down-regulation of photosynthesis in both FACE studies (Ainsworth and Long, 2005) and chamber experiments (Warren et al., 2014). This occurrence is known as photosynthetic acclimation. Photosynthetic acclimation, however, does not always completely negate the positive effects e[CO_2_] has on photosynthesis. For example, in one study white clover was grown under elevated (600 ppm) [CO_2_] for 8 years and retained a 37% increase in photosynthesis after acclimation was observed (Ainsworth et al., 2003). These findings suggest that final growth response to e[CO_2_] is largely determined by the magnitude of plant acclimation to e[CO_2_].

Various explanations as to the cause of photosynthetic acclimation have been made. Decreased leaf nitrogen (N) is one such explanation. In a study on rice, e[CO_2_] caused a decline in N allocation into leaf blades, which subsequently reduced Rubisco and other protein synthesis (Seneweera et al., 2011). In support of this, in a 12 year study on *Liquidambar styraciflua* no acclimation response occurred during the time when leaf N was sufficient for photosynthetic requirements (Warren et al., 2014). Without sufficient N to invest in Rubisco, the photosynthetic capacity of the leaf declines. Low availability of soil nitrate increases the severity of photosynthetic acclimation and seems also to be associated with an inhibition of leaf nitrate assimilation (Vicente et al., 2016). Inhibition of leaf nitrate assimilation also occurs under e[CO_2_] (Bloom et al., 2014). It is not known whether the reduction of Rubisco synthesis at e[CO_2_] is directly related to lower N assimilation or if Rubisco is just regulated to balance the source and sink activity.

Another explanation for plant acclimation to e[CO_2_] is that an increase in sugar production tips the source—sink balance of plants, potentially resulting in more sugars being produced in source tissues than can be utilized in sink tissues. This was the case in a FACE experiment by Ainsworth et al. (2004), who used single gene mutations to test the hypothesis that photosynthetic acclimation is due to inadequate sink capacity. In the study, a soybean cultivar with an indeterminate growth trait (Williams) was compared with a line mutated for determinate growth (Williams-*dt1*). Only the determinate line showed photosynthetic acclimation. On the other hand, mutation of a determinate soybean cultivar (Elf) to an indeterminate form showed no increased photosynthesis. While this may provide evidence for single gene mutations being responsible for photosynthetic acclimation, this could also be explained by the fact that Elf is a cultivar bred to avoid sink limitations (Ainsworth et al., 2004). While sink capacity remains high, plants are able to continue to utilize the greater CO_2_ availability. However, with limited carbon sink capacity the plant must decrease photosynthesis in order to maintain source activity. As such, when e[CO_2_] causes photosynthesis to surpass what the plant is capable of utilizing or exporting to sinks, an accumulation of non-structural carbohydrates (NSC) occurs (Ainsworth et al., 2004) and leads to feedback inhibition of photosynthesis (Figure 1).

These NSCs are then able to affect gene transcription through their role as signaling molecules (Mishra et al., 2009; de Jong et al., 2014). As such, sugars are known to be involved in photosynthetic acclimation, whereby the extra carbohydrates produced under e[CO_2_] cause a down-regulation of photosynthetic gene transcripts and suppress protein synthesis, thereby decreasing the rate of photosynthesis (Cheng et al., 1998). In this way, there is a feedback inhibition where the products of photosynthesis cause suppression of photosynthesis, leading to photosynthetic acclimation.

Rubisco, an essential enzyme in the photosynthetic pathway, is known to be decreased in leaves that have an accumulation of carbohydrates (Cheng et al., 1998; Aranjuelo et al., 2008). Despite this evidence, a study by Ludewig and Sonnewald (2000) opposed the hypothesis that accumulation of sugars leads to photosynthetic acclimation when they found that high [CO_2_] caused accelerated leaf senescence in *Nicotiana tabacum*, leading to down-regulation of leaf photosynthetic related genes and thus accelerated leaf senescence. Only senescing leaves were found to show down-regulation of photosynthetic genes and increased sugar levels were not observed. They concluded that photosynthetic acclimation was caused by leaf senescence rather than sugar accumulation. Both Aranjuelo et al. (2008) and Cheng et al. (1998), however, reported that the down-regulation of photosynthesis occurred prior to senescence of the plants. All three studies used different plant species, which suggests that some species acclimate to e[CO_2_] differently than others. Therefore, this suggests that photosynthetic acclimation has no single cause, with multiple processes each contributing to a different degree.

## Effect of elevated [CO_2_] on carbohydrate biosynthesis and allocation between organs

As discussed in the previous section, e[CO_2_] causes an increase in carbohydrate production via the stimulation of photosynthesis. It has been observed that increased photosynthesis under e[CO_2_] results in greater production of certain carbohydrates compared to others. The concentration of sucrose, the main product of photosynthesis, increases in all organs of pea plants exposed to e[CO_2_] in growth chambers, however, glucose concentrations are largely unaltered (Aranjuelo et al., 2013). Glucose measurements may be inaccurate as glucose content can fluctuate throughout the day in some plants, increasing and then decreasing as the day progresses (Seneweera et al., 1995; Grimmer et al., 1999). As such, hexose to sucrose ratio will differ depending on what time period the glucose levels are measured. Glucose measurements taken when glucose levels are naturally low, will give a lower hexose to sucrose ratio than if glucose was measured during a period of high glucose levels. Sucrose levels also increased in castor oil plants grown in growth chambers under 700 ppm CO_2_ compared to 350 ppm, increasing by an average of one third (Grimmer et al., 1999). Levels of sucrose are higher than that of hexoses under e[CO_2_] in both chamber and field studies (Grimmer et al., 1999; Rogers et al., 2004), however, in soybean the leaf hexose-carbon to sucrose-carbon ratio increases with exposure to e[CO_2_], where a five-fold greater ratio of hexose-carbon to sucrose-carbon was observed near the end of the growing season (Rogers et al., 2004). Perhaps, such variation in hexose to sucrose ratio during plant development may affect plant source and sink activities. In addition, the preference of a plant to produce one type of carbohydrate over another could potentially be linked to the control of genes by a specific carbohydrate (glucose, sucrose, etc.), though this is not known. For example, if a plant requires the presence of sucrose to initiate the repression of a specific gene, it would be ineffective to produce greater glucose quantities than sucrose. The effect that carbohydrates have on gene expression is a topic discussed further in this review, however, the impact that a change in sugar composition has on plant gene regulations is not well understood.

Starch, a major storage carbohydrate in plants, is also increased in plants growing in e[CO_2_] (Aranjuelo et al., 2008). The increase in starch likely contributes to the high levels of sucrose observed with e[CO_2_], due to the conversion of starch to sucrose overnight. This conversion is important for normal plant growth under ambient conditions (Smith et al., 2005), however, under e[CO_2_] it may contribute to the accumulation of sucrose. In plants grown under ambient [CO_2_] the starch content builds up during the day and disappears overnight. The increased production of starch under e[CO_2_], however, means that not all of the plant's starch reserves are depleted during the night, leading to a gradual accumulation in leaves over time (Grimmer et al., 1999). Different plant species accumulate different amounts of sucrose compared to starch, for example spinach accumulates more sucrose and cotton more starch (Goldschmidt and Huber, 1992). These responses are likely to affect the sugar sensing pathways in either type of plant. The degree of carbon partitioning between sucrose and starch is influenced by the length of daylight. In shorter periods of light, carbon partitioning shifts toward starch synthesis, while sucrose synthesis and consumption is decreased (Pokhilko et al., 2014). Less starch is accumulated during days with long light periods, while sucrose synthesis is increased (Pokhilko et al., 2014). Sucrose content is greater during the day than night, but the amount of sucrose remaining at the end of the day, as well as the end of the night, decreases as day length decreases (Sulpice et al., 2014). The degradation of starch at night is influenced by the amount of trehalose-6-phosphate (T6P). Increased T6P was found to inhibit starch degradation at night in *Arabidopsis* plants, resulting in much higher starch reserves at the end of the night (Martins et al., 2013). In addition, Martins et al. (2013) found that T6P also slightly increases starch synthesis. As such, increased T6P concentrations result in more starch at both the end of the day and night. Combined with limitations on starch degradation set by the plant's circadian clock, these findings suggested a model for overnight starch metabolism (Martins et al., 2013; Lunn et al., 2014). High sucrose demand causes lower T6P, alleviating the inhibition of starch degradation and increasing sucrose content. Under low sucrose demand, T6P increases and inhibits starch degradation. The plant's circadian clock prevents the total depletion of starch at night by setting limits on starch degradation based on the length of the night period (Martins et al., 2013).

The extra carbohydrates that accumulate in leaves are allocated to the rest of the plant in varying amounts, where some organs receive more of these carbohydrates than others. Little research has been done into the allocation of carbohydrates under e[CO_2_], but the following studies have investigated this. Carbohydrate allocation under e[CO_2_] varies with species. Some species allocate more carbon to the seeds and others to the shoots, leaves or roots (Salsman et al., 1999; Sasaki et al., 2007; Aljazairi et al., 2014; Butterly et al., 2015). For example, during the grain filling stage of rice e[CO_2_] promotes the translocation of carbohydrates stored in vegetative tissues to the panicle, as well as allocating newly fixed carbohydrates to the panicle, where it is stored as starch (Sasaki et al., 2007). A difference in carbon allocation between durum wheat and bread wheat occurs under e[CO_2_]. Durum cultivars Blanqueta and Sula allocated more carbon into roots, rather than shoots (Aljazairi et al., 2014), while the bread wheat cultivar Yitpi allocated more carbon into shoots (Butterly et al., 2015). Furthermore, Sula (a modern cultivar) allocated more carbon into spikes compared to Blanqueta (a traditional cultivar), which allocated more carbon into non-reproductive shoot tissue. This indicates that variation exists within as well as between species and suggests that genetics contributes to these differences. In the case of the two durum cultivars, both differed in yield potential. Sula, which invested more carbon in spikes, is a higher yielding wheat than Blanqueta. Elevated [CO_2_] also increased growth of roots and shoots of tepary bean, where the roots saw a ten-fold increase in starch (Salsman et al., 1999). Allocating more carbon into roots under e[CO_2_] would contribute to greater root growth, allowing improved nutrient and/or water uptake and thus would help to maintain the balance of nutrients within the plant.

Carbon dioxide concentration is not the sole regulator of carbohydrate partitioning, with many other environmental factors involved in shaping the outcome. Which carbohydrate the increased carbon is partitioned into can be affected by the method plants use to take up nitrogen. An experiment by Aranjuelo et al. (2013) found N_2_-fixing and ${\text{NO}}_{3}^{-}$-fed plants varied greatly in sucrose content while exposed to e[CO_2_]. Sucrose increased by 366% in ${\text{NO}}_{3}^{-}$-fed plants but only by 56% in N_2_-fixing plants. As e[CO_2_] is known to affect the uptake and assimilation of N in plants (Bloom et al., 2014; Vicente et al., 2015a), this could point to a link between N uptake and carbohydrate allocation to roots and thereby facilitating more nutrient uptake. Plant growth method (glasshouse, field, etc.) also affects carbon allocation. Elevated [CO_2_] causes increased carbon allocation to roots of perennial rye-grass resulting in increased root dry matter when grown in field conditions, however, no such results occur when grown in controlled environment chambers (Suter et al., 2002). This outcome in rye-grass was attributed to a difference in N availability, plant age and shoot sink strength. Results from Aranjuelo et al. (2013) also indicate that sink strength affects carbon allocation, where increased carbon sink strength of N_2_-fixing plant's nodules allows greater storage of carbohydrates which in turn prevents the inhibition of photosynthesis by increased carbohydrates. This could mean that control of carbon allocation could be partially affected by the availability of carbon sinks. Another factor that may affect the allocation of carbohydrates under e[CO_2_] is the effect e[CO_2_] has on leaf area, as appeared to be the case for N allocation in rice (Makino et al., 1997). Plants which show less variable responses to leaf area under e[CO_2_] (e.g., rice; Makino et al., 1997) compared to others, may allocate more carbohydrates to roots, as their leaf sink capacity doesn't change to accommodate the greater carbohydrate production. For some plants, root growth is increased under e[CO_2_] (George et al., 2003), which may increase their sink capacity, allowing for greater allocation of carbohydrates to this organ. Carbon allocation under e[CO_2_] can also be influenced by pH, as seen in plants grown in a low pH media under e[CO_2_], where much of the carbon from photosynthesis accumulates in the shoots (Hachiya et al., 2014).

## Sugar sensing and signaling: an overview

There are many reviews already written on the role of sugars as signals in plants including Granot et al. (2013), Rolland et al. (2006), and Sheen (2014) to name a few. However, to the best of our knowledge there are no reviews written specifically for sugar sensing in roots, which is a major focus of this review. As such, before moving on to our discussion of sugar sensing in roots, this section will serve to provide general information on sugar sensing not specific to roots. There is much more information known on sugar sensing than written in this section, however, we direct you to other reviews, such as those mentioned above, for more detailed discussions on sugar sensing not specific to roots.

Glucose has long been known to play a role in photosynthetic gene repression, with the enzyme hexokinase acting as a sensor (Jang and Sheen, 1994). It has since been established that hexokinase is a central enzyme in glucose sugar signaling pathways (Moore et al., 2003). Through sugar sensing, hexokinase appears to be able to promote plant growth by causing greater cell expansion in roots, leaves, and inflorescences when exposed to high light conditions (Moore et al., 2003).

In addition to hexokinase, SnRK1 has been indicated as another sugar sensor which is involved in a sucrose/T6P signaling network and operates as a starvation response (Baena-Gonzalez et al., 2007). It has been observed that SnRK1 may be inhibited by the presence of sucrose. KIN10, a part of the SnRK1 complex, is activated under sugar starvation, leading to up-regulation and down-regulation of various genes (Baena-Gonzalez et al., 2007). SnRK1 also contributes to increasing sugar content in plants by phosphorylating both sucrose phosphate synthase (SPS) and trehalose-phosphate synthase (TPS; Nukarinen et al., 2016), of which the resulting sugars, sucrose and T6P, may lead to inactivation of SnRK1 (Baena-Gonzalez et al., 2007; Zhang et al., 2009). Sucrose concentrations are linked with T6P levels, as increased sucrose leads to stimulation of TPS which in turn increases T6P concentrations (Yadav et al., 2014). High T6P then causes a decline in sucrose content which prevents further increases in T6P (Yadav et al., 2014). The regulation of T6P content is primarily linked with sucrose content, as studies have shown that only sucrose and hexoses able to be converted to sucrose have a significant effect on T6P levels (Lunn et al., 2006; Yadav et al., 2014). Sucrose and T6P may also be involved together with nitrogen assimilation, where increases in T6P signal the plant to synthesize organic and amino acids rather than sucrose (Figueroa et al., 2016). In conjunction with T6P other similar sugar phosphates, glucose 1-phosphate (G1P) and glucose 6-phosphate, are able to inhibit SnRK1, with G1P working together with T6P to significantly increase this inhibition (Nunes et al., 2013). Altogether SnRK1 appears to be involved in the plant's starvation response, inactivating during times of sufficient sucrose/T6P and activating when these signals are low.

Sugar signaling in plants begins as early as seed development and germination. At low levels, sugars are able to delay germination of *Arabidopsis thaliana* seeds. Other sugars have displayed this function as well, with sucrose, glucose, and the non-metabolically active glucose analog 3-O-methyl glucose exhibiting a greater delay on germination than others (Dekkers et al., 2004). The ability of the glucose analog to delay germination indicates a pathway independent of hexokinase.

Sucrose functions as a signaling molecule in a variety of ways. It is capable of inducing gene expression, such as, the *Citrus* ammonium transporter gene *CitAMT1* (Camañes et al., 2007), as well as affecting the cell cycle. During the G1 phase of the cell cycle, sucrose induces the expression of the two CycD cyclins *Cyc2* and *Cyc3*, which influence cell cycle progression and cell division (Riou-Khamlichi et al., 2000). The role of sucrose in regulating the cell cycle likely correlates with its role in plant growth. As a plant produces more sugars, sucrose stimulates the cell cycle and allows utilization of the produced sugars for growth. As such, e[CO_2_] is likely to facilitate this process. The greater sugar production caused by e[CO_2_] could stimulate the cell cycle and allow the excess sugars to be used to produce greater plant biomass (Seneweera and Conroy, 2005).

Sugar signaling pathways also interact with hormones. For example, glucose increases the biosynthesis of auxin, therefore affecting processes regulated by this hormone (Sairanen et al., 2012). Evidence also suggests that sugars interact with pathways of both abscisic acid (Cheng et al., 2002) and ethylene (Price et al., 2004). Among other functions, abscisic acid has an enhancing effect on some genes regulated by sugar (Rook et al., 2001), while glucose downregulates the expression of ethylene biosynthetic genes (*VnACO2* and *VnEIL1*) and a transcription factor involved in the ethylene signaling pathway of narbon bean cotyledons (Andriunas et al., 2011). These findings show the various roles of sugars in gene regulation and thus their contribution to plant growth and development by way of sugar sensing.

## Sugar sensing and signaling in roots

Currently there is a lack of understanding about the effect of e[CO_2_] on sugar sensing, however, many studies have conducted experiments applying exogenous carbohydrates to plant roots, thus creating conditions of increased root sugar content which may mirror the conditions of greater root sugar content resulting from increased photosynthesis under e[CO_2_]. Most of the research into the role carbohydrates play in plant roots has focussed on sucrose exclusively. While some research has brought to light several effects of other carbohydrates, such as, glucose and fructose, there may yet be many more roles that non-sucrose carbohydrates play. Much of this work is limited to *A. thaliana*, but it is likely that sugars play many other diverse roles in root function that may be discovered among other plant species. The following section discusses the potential outcomes for roots of plants grown under e[CO_2_], whereby excess carbohydrates in leaves are transported to roots and lead to altered gene expression (Figure 1). The effects of sugar sensing in roots has had less attention then in shoots, as is especially the case for sugar sensing under e[CO_2_]. As such, there is insufficient data to draw conclusions at this time, however, we provide an insight into how e[CO_2_] may affect sugar sensing in roots, as well as sugar crosstalk with hormones.

### Sugar sensing and gene expression in roots

${\text{NO}}_{3}^{-}$ uptake is diurnally regulated in a variety of plants (Lejay et al., 1999; Ono et al., 2000; Feng et al., 2011). In *A. thaliana* the ${\text{NO}}_{3}^{-}$ transport genes *Nrt2.1* and *Nrt1*, which are down-regulated at night, are induced by sucrose application at night (Lejay et al., 1999), a result also seen with rice *Nrt2* genes (Feng et al., 2011). This could mean that if sugars accumulate in roots of e[CO_2_] grown plants during the night, the diurnal cycle of ${\text{NO}}_{3}^{-}$ transport will be affected. In plants that store starch in their roots, this could lead to an accumulation of sucrose in roots throughout the night, leading to altered gene transcription overnight. Sucrose concentration is also responsible for transcriptional regulation of other diurnally-regulated root ion transporters. Sucrose regulates three ${\text{NH}}_{4}^{+}$ transporters (*AtAmt1.1, AtAmt1.2*, and *AtAmt1.3*), an ${\text{SO}}_{4}^{2-}$ transporter (*AtHst1*), a phosphate transporter (*AtPt2*), a K^+^ transporter (*AtKup2*), a metal transporter (*AtIrt1*), and a K^+^ channel (*AtSkor*), though each to a different degree (Lejay et al., 2003). Sucrose also contributes to regulation of ammonium uptake in *Citrus* plants, via stimulating expression of *CitAMT1* (Camañes et al., 2007). Though sucrose has the ability to regulate root ion transporters, they are not all regulated by the same mechanism. Lejay et al. (2008) found that three different signaling pathways regulated the expression of 16 sugar-induced root ion transporters. Most genes (ten) appeared to be regulated by a pathway dependent on the catabolic activity of hexokinase, rather than its sensing function, whereby the downstream metabolites of glycolysis act as signals for gene regulation. A second pathway, affecting five genes, involved a sucrose and/or glucose signal prior to hexokinase activity. Hexokinase sensing was proposed as the third pathway, which affected a single gene. All three pathways are briefly reviewed in Rolland et al. (2006) where they are referred to as the glycolysis-dependent pathway, HXK1-independent signaling pathway, and HXK1-dependent pathway, in order of those mentioned above. Among these genes, the majority appeared to also respond to [CO_2_] (Lejay et al., 2008). If no sucrose was applied exogenously to the plants, 11 of the 16 genes responded to light exposure, provided there was also CO_2_ in the atmosphere. In addition to this, ten of the genes were observed to respond further at higher [CO_2_] (600 μL L^−1^ CO_2_) rather than low [CO_2_] (300 μL L^−1^ CO_2_). This may suggest that these genes display a varied response depending on the amount of photosynthate produced. As such, these results may support our argument that greater photosynthesis caused by e[CO_2_] will change the level of expression of some genes in roots.

Sucrose can also stimulate nitrogen assimilation via the oxidative pentose phosphate pathway (OPPP). An increase in sucrose concentration in roots of *A. thaliana* causes the induction of OPPP genes (*G6PDH2, G6PDH3, 6PGDH2*) and nitrate/nitrite reduction genes (*NIA1, NIA2, NiR;* Bussell et al., 2013). This induction requires plants to have a functional plastidial OPPP, which suggests that sucrose influences the OPPP to produce a signal that leads to transcription of N assimilation genes (Bussell et al., 2013). Not only is the OPPP important for sucrose mediated nitrogen assimilation, but it is also required for glucose mediated *Nrt2.1* expression. Glucose affects the OPPP via HXK1, which ultimately leads to the stimulation of *Nrt2.1* transcription (de Jong et al., 2014). Glucose also appears to post-transcriptionally regulate Nrt2.1 protein levels and transport, however, this appears to be independent of the mechanism used to stimulate *Nrt2.1* transcription via HXK1 (de Jong et al., 2014). Utilization of the *glucose-insensitive2-1* (*gin2-1*) mutant, which lacks the hexokinase sugar sensing mechanism, showed that glucose regulates *Nrt2.1* transcription independently of nitrate-mediated regulation (de Jong et al., 2014). However, it is not known how these genes function under dynamic changes to sugar composition at e[CO_2_]. There is evidence that transcription of OPPP genes in leaf tissue is down-regulated under e[CO_2_] (Vicente et al., 2015b), but there was no evidence to suggest sugars as the cause of the down-regulation. Given that sucrose and glucose can affect the OPPP in roots, it is reasonable that a similar system may exist in leaf tissue. The down-regulation seen in Vicente et al. (2015b) may then be attributable to increased sugar production under e[CO_2_]. As increased sucrose in roots cause induction of OPPP genes, an increase in sucrose due to increased photosynthesis under e[CO_2_] may cause a similar interaction in leaves, but down-regulating the genes instead.

Sugars may also contribute to nutrient uptake by control of genes involved in root formation. Sucrose regulates the gene *CYCD4;1*, a member of the D-type cyclins (De Veylder et al., 1999) which belongs to a family of proteins, called cyclins, that regulate cell cycle progression (Mironov et al., 1999). The cyclin *CYCD4;1* is expressed in pericycle cells of the root apical meristem and is involved in lateral root primordia formation (Nieuwland et al., 2009). This may be, in part, how sugars are able to regulate root growth, as discussed in the next section. In addition, this may explain one way that e[CO_2_] is able to increase root growth (Lee-Ho et al., 2007).

Sugars are important regulators in phosphate deficient plants. During phosphate starvation, carbohydrates are used to regulate various phosphate starvation induced (PSI) genes (Karthikeyan et al., 2007). Glucose and fructose can stimulate PSI genes to an extent, however, optimal responses occur with sucrose. During phosphorus deficiency, sucrose is able to increase the expression of a phosphate transporter gene (*LaPT1*) and a phosphoenolpyruvate carboxylase gene (*LaPEPC3*; Zhou et al., 2008). Sucrose also promotes growth of root hairs in phosphate deficient *A. thaliana* (Jain et al., 2007). The increased sugar production under e[CO_2_] likely leads to lower inorganic phosphorus in plants due to the use of phosphorus in sugars such as triose phosphate, the synthesis of which will likely increase under e[CO_2_]. The lower phosphorus concentration then becomes limiting in ATP synthesis and regeneration of ribulose bisphosphate (Farquhar and Sharkey, 1982). Whether the increased sugar production under e[CO_2_] provokes the same expression of PSI genes mentioned above, is not currently known. Research has shown that e[CO_2_] increases the expression of the phosphate uptake gene *AtPHR1* in phosphate deficient *Arabidopsis* plants (Niu et al., 2013), however, more research is needed to elucidate the role of e[CO_2_] in sugar mediated PSI gene regulation.

There may be many genes in the root that are unrelated to nutrient acquisition which are activated by a sugar signal. For example, almost every aspect of auxin metabolism appears to be affected or regulated by glucose. Out of 604 auxin regulated genes in *A. thaliana*, 376 (62%) are transcriptionally regulated by glucose, which range in function from the biosynthesis of auxin to its transport, perception, and signaling (Mishra et al., 2009). Amino acid synthesis may also be impacted by sugar sensing. Silvente et al. (2008) found that glucose, acting through hexokinase, increased production of asparagine synthetase in roots of common bean. This brings to light more ways that e[CO_2_] could affect root processes through sugar sensing. Research needs to be conducted in this area before any conclusions can be drawn, however, given that e[CO_2_] has been shown to affect sugar regulation of genes in roots (Lejay et al., 2008), these findings show there is potential to find that auxin metabolism and amino acid synthesis can also be regulated to some extent by e[CO_2_] through sugar sensing.

Under e[CO_2_] conditions, Jauregui et al. (2015) found that expression of 48 genes of various functions, including genes linked with photosynthesis, hormones, and stress, was affected in *A. thaliana* roots, 95% of which were downregulated. The main finding of this study, however, showed that supplying e[CO_2_] treated *A. thaliana* plants with ammonium nitrate improved plant protein content and maintained higher photosynthetic rates. This suggests that altering the nitrogen availability of plants may affect the plant's sugar sensing capabilities, as altering the plant's photosynthetic capacity will ultimately alter the carbohydrate content of plants. The mechanism by which e[CO_2_] affected the 48 genes was not explored in the paper and as such, we don't know whether they were affected via sugar sensing pathways. The sugar content of the roots under e[CO_2_] did not differ significantly from roots of plants grown under ambient [CO_2_], however, there was a slight increase in sucrose content. Whether this small increase is enough to alter gene expression in roots is uncertain. Another possibility is that faster sugar catabolism may promote gene expression, however, the process is totally unknown and more research into the effect of e[CO_2_] on gene expression in roots is required. Lower nutrient concentration in grains has been widely reported under e[CO_2_] (Taub et al., 2008; Högy et al., 2013; Fernando et al., 2015), but whether these declines are associated with sugar mediated gene expression causing altered nutrient assimilation is unknown.

### Elevated [CO_2_] and sugars affect root architecture

Storage of the accumulated carbon under e[CO_2_] is not consistent across all plants. In some plants, e[CO_2_] causes a shift in the shoot/root carbon ratio toward greater root carbon (Aljazairi et al., 2014). How this extra carbon affects roots is not well understood, however, understanding the extent that sugars affect roots will provide a starting point for research into the effect of e[CO_2_] on roots.

Elevated [CO_2_] has a similar effect on root growth as increased sucrose concentrations. This may suggest that the way in which e[CO_2_] affects root growth is through the increased sugars allocated to roots. Elevated [CO_2_] increases both total root number and length in *A. thaliana* as well as root diameter (Lee-Ho et al., 2007). Increasing sucrose concentration in plants grown under ambient [CO_2_] also gives results similar to e[CO_2_] (Lee-Ho et al., 2007). Elevated [CO_2_] may increase root growth in order to balance nutrient uptake with the rate of sugar production from increased photosynthesis or perhaps a larger root system acts as a sink to store excess sugars.

Gaining a better understanding of how e[CO_2_] affects the growth of roots could help explain the changes in nutrient status that occur under e[CO_2_], such as the deficiencies of iron and zinc in wheat (Myers et al., 2014). With both e[CO_2_] and sugars increasing plant root growth, you would expect greater uptake rates of nutrients, thus relieving nutrient deficiencies. While there are other mechanisms that are affected by e[CO_2_] that lead to nutrient deficiencies, their discussion is outside the scope of this review. The role that roots play in causing or alleviating nutrient deficiencies needs to be further elucidated.

The carbohydrate status of plants can strongly influence root architecture. For example, increasing concentrations of the hexoses glucose and fructose in the growing regions of *A. thaliana* roots are positively correlated with both root elongation rate and branching density (Freixes et al., 2002). Not all hexoses work to promote root elongation, however, as mannose inhibits root elongation by a signaling pathway initiated by hexokinase (Baskin et al., 2001). Galactose, another hexose, also inhibited root elongation in the study by Baskin et al. (2001), but to a lower extent. Psicose, an analog of fructose, is a third hexose capable of inhibiting root growth. It was found to inhibit root growth of lettuce seedlings, however, in contrast with mannose, it does not appear to cause the inhibition through a hexokinase-mediated pathway (Kato-Noguchi et al., 2005). Elevated [CO_2_] generally increases root growth in FACE and open-top chambers (OTC; Milchunas et al., 2005; De Graaff et al., 2006). In *Sedum alfredii*, e[CO_2_] is found to increase both root elongation and branching (Li et al., 2012), while other studies have found a variety of plant species show increased fine root production (Pritchard and Rogers, 2000; Tingey et al., 2000). A meta-analysis of FACE and OTCs found a general increase in root biomass in response to e[CO_2_], where root length was increased more than root diameter (Nie et al., 2013). The meta-analysis also found that increased fine root biomass was the main component of the total biomass increase. This may suggest that if e[CO_2_] plays a role in the sugar stimulated increase in root growth, more carbon is partitioned into sugars such as glucose, which is capable of increasing root growth, rather than psicose or mannose. Therefore, understanding how diurnal changes in sugar composition is affected under e[CO_2_] will provide a greater insight into the role that sugars have on root growth and gene expression in response to e[CO_2_].

The role of glucose in *A. thaliana* roots is not limited to root elongation rate and branching density. It has also demonstrated the ability to control root growth direction in *A. thaliana*, and it does this independently of changes in root length (Singh et al., 2014b). The directional change induced by glucose occurs via both hexokinase dependent and independent methods (Singh et al., 2014b). The hexokinase glucose sensing pathway also leads to increased lateral root production (Gupta et al., 2015). Furthermore, root meristem activation is stimulated by glucose via a target-of-rapamycin (TOR) signaling network (Xiong et al., 2013). The control of root meristem activation by the glucose-TOR interaction relies on glycolysis–mitochondrial energy relays. This signal network in turn promotes root growth.

Sucrose has been identified as a necessary signal to stimulate primary root growth in *A. thaliana* seedlings, where the sucrose is transported to the roots from the cotyledons by way of the sucrose transporter SUC2 (Kircher and Schopfer, 2012). In addition, secondary root growth is also promoted by sucrose (Freixes et al., 2002). Sucrose also has the ability to rescue plants from certain factors which inhibit root growth. The inhibition of root growth caused by both psicose and mannose, as previously mentioned, is overcome by the addition of sucrose (Kato-Noguchi et al., 2005). This means that in plants that produce more sucrose under e[CO_2_] than hexoses, the inhibition by psicose and mannose is unlikely to occur.

As previously mentioned, sucrose is also involved in promoting lateral root primordia formation, however, Macgregor et al. (2008) argues that this regulation is caused by the metabolism of sucrose, rather than sucrose acting as a signal. They concluded this on the basis that sucrose and its downstream metabolites glucose, fructose, and glucose-6-phosphate, all promoted lateral root primordia formation, but the non-metabolized glucose analog 3-*O*-methyl glucose did not, combined with the observation that exogenous sucrose promoted lateral root primordia formation in the hexokinase mutant *gin2*. It could instead be argued that these sugars operate as signals independently of hexokinase, particularly sucrose which is not sensed by hexokinase. Despite evidence that sugars promote lateral root development, a recent study concluded that sucrose and glucose promote the expression of the *A. thaliana WOX7* gene, which inhibits lateral root growth (Kong et al., 2016). Adding to the complexity surrounding the influence of sugars on regulatory pathways, auxin, a hormone that promotes lateral root development and is upregulated by sugars, represses *WOX7* expression (Kong et al., 2016).

Further aspects of the ability for sugars to control root architecture are discussed in the next section, where crosstalk with various plant hormones is required to bring about changes in root architecture.

### Elevated [CO_2_] mediates sugar and hormone crosstalk

Along with the ability for sugars to control gene expression and root growth, they also are known to interact with hormones, extending their potential effect as a signaling molecule. For instance, sucrose-mediated induction of the *Nrt* gene may be due to its capability to crosstalk with auxin, a hormone which, among other functions, regulates the *A. thaliana* nitrate transport gene *AtNrt1.1* (Guo et al., 2002). Exogenously introduced auxin stimulates *AtNrt1.1* transcription at the commencement of lateral root formation (Guo et al., 2002). In addition to crosstalk with auxin, sucrose stimulates both auxin production and transport to roots (Lilley et al., 2012). Glucose and sucrose are able to regulate the biosynthesis of the auxin called indole-3-acetic acid (IAA), though sucrose has a greater effect on IAA biosynthesis (Sairanen et al., 2012). As such, by regulating the production and transport of auxin, sugars are indirectly influencing the plant processes brought about by auxin. Auxin also works with glucose to promote formation of lateral roots in *A. thaliana*. In the presence of glucose, the formation of auxin-induced lateral roots is bimodal, where the number of lateral roots peaks at both low and high concentrations, but not medium (Booker et al., 2010). Glucose acts to inhibit the heterotrimeric G protein complex, which attenuates this bimodality (Booker et al., 2010). Auxin stimulates the cell cycle to promote lateral root initiation and also affects the frequency and position of lateral roots, depending on the amount of auxin and the direction of its flow in the roots (Himanen et al., 2002). This may contribute in part to glucose's ability to promote lateral root growth, as discussed in the previous section, however, this is unknown. Glucose can also cause root hair initiation and elongation, however, elongation is decreased in the absence of auxin (Mishra et al., 2009).

Glucose interacts with another hormone, brassinosteroid, to stimulate lateral root formation. Brassinosteroid works downstream of the HXK1 glucose sensing pathway (Gupta et al., 2015). This glucose and brassinosteroid mediated pathway also affects auxin transport machinery during lateral root production (Gupta et al., 2015), thus contributing to the auxin-mediated lateral root formation. Brassinosteroid also works with glucose to control root growth direction (Singh et al., 2014b). It appears that polar auxin transport is also involved in glucose induced root growth direction, occurring downstream from glucose and brassinosteroid (Singh et al., 2014b). Working antagonistically to this control of root growth direction, however, are ethylene and cytokinin, which, together with glucose, brassinosteroid and auxin, may make up a system for controlling the growth direction of plant roots (Singh et al., 2014a). Exposure of *A. thaliana* root tips to the hormone cytokinin promotes root growth via cell elongation (Kushwah et al., 2011). This root growth is further promoted by the presence of glucose which operates through hexokinase.

There is limited research focusing on the relationship between e[CO_2_] and plant hormones, however, several studies have shown the effect of e[CO_2_] on hormone synthesis. Results from Hachiya et al. (2014) suggest that e[CO_2_] can cause preferential root growth by increasing root IAA content. Increased sugar production under e[CO_2_] appears to cause increased biosynthesis of IAA in shoots, which is subsequently transported to roots. That both sucrose and glucose are known to stimulate IAA biosynthesis in roots under ambient [CO_2_] could suggest that this is the mechanism used to cause the increase under e[CO_2_]. Auxin and sugars also appear to work together in roots of iron (Fe)-deficient plants. A recent study proposed a model whereby Fe-deficiency increases sucrose content of roots, causing an increase in auxin and a subsequent increase in nitric oxide, ultimately causing FIT-mediated transcriptional regulation of *FRO2* and *IRT1* genes and inducing Fe uptake (Lin et al., 2016). If these genes are regulated by the increase of sucrose, then it stands to reason that an increase in sucrose content in roots brought about by e[CO_2_] might bring about the same change. In a hydroponics study, IAA content in roots was increased by e[CO_2_] in tomato plants by 26.5% (Wang et al., 2009). IAA was not the only hormone increased by e[CO_2_]. They also found ethylene release in roots was increased by 100% in tomato plants when grown under e[CO_2_], showing that stimulation of hormone production under e[CO_2_] is not limited to auxin. Ethylene was also found to be increased in rice plants grown in growth chambers under e[CO_2_] (Seneweera et al., 2003). In addition to auxin and ethylene, jasmonic acid has also been reported to be regulated under e[CO_2_]. However, as opposed to the stimulation of auxin and ethylene seen in other studies, the synthesis of jasmonic acid was repressed by e[CO_2_] in Guo et al. (2012). This was, however, found to occur in leaves. Whether e[CO_2_] affects jasmonic acid in roots is unknown. That both e[CO_2_] and sugars have been demonstrated to interact with plant hormones may suggest that in future climates, the sugars produced under e[CO_2_] may act as intermediates for hormonal crosstalk.

## Concluding remarks

Much is still unknown about how plants will react to e[CO_2_] and with nutrient deficiencies observed in agricultural crops, this will become increasingly more important to understand. The production of carbohydrates is increased in plants grown under e[CO_2_] due to an increase in photosynthesis. Some carbohydrates are produced in higher quantities than others depending on the plant, though production of sucrose is reportedly higher compared to hexoses. The studies discussed provide an insight into how these sugars can be used to regulate many functions in roots. Most of the information on sugar signaling discusses the glucose and sucrose pathways. The amount of carbon partitioned into either of those carbohydrates may be in part determined by which carbohydrate the plant requires to regulate specific genes, though this is unknown. Nutrient acquisition appears to be regulated by sugars, as evidenced by the regulation of expression of various ion transporters as well as the ability for sugars to affect root growth. Finally, both e[CO_2_] and sugars are able to affect the biosynthesis of certain plant hormones, which may suggest that sugars function as an intermediate in e[CO_2_] control of hormones. From these studies we can begin to think about what changes might occur in roots of plants grown in future carbon dioxide concentrations.

## Author contributions

MT wrote the manuscript. SS, AM, NH, and DG each contributed to the design of the review as well as revision of drafts and the final manuscript.

### Conflict of interest statement

The authors declare that the research was conducted in the absence of any commercial or financial relationships that could be construed as a potential conflict of interest.
